# In-hospital survival of adults with HIV-associated cryptococcal meningitis in Tanzania: A retrospective comparison of amphotericin B-based regimen and fluconazole monotherapy

**DOI:** 10.1371/journal.pone.0332786

**Published:** 2025-12-05

**Authors:** Mlela Msongela, George Musiba, Juma A. Mohamed, Alphonce I. Marealle, Manase Kilonzi, Ritah F. Mutagonda

**Affiliations:** 1 Department of Pharmacy, Singida Regional Referral Hospital, Singida, Tanzania; 2 Department of Clinical Pharmacy and Pharmacology, School of Pharmacy, Muhimbili University of Health and Allied Sciences, Dar es Salaam, Tanzania; 3 Department of Pharmacy, Muhimbili National Hospital, Dar es Salaam, Tanzania; 4 Department of Pharmaceutics and Pharmacy Practice, School of Pharmacy, Muhimbili University of Health and Allied Sciences, Dar es Salaam, Tanzania; Gulu University, UGANDA

## Abstract

**Objective:**

This study aimed to examine treatment modalities, outcomes, and factors associated with in-hospital survival among people with HIV (PHIV) diagnosed with cryptococcal meningitis (CM) in Tanzania.

**Methods:**

This hospital-based cross-sectional study retrospectively reviewed records of PHIV admitted to medical wards at Dodoma and Singida Regional Referral Hospitals in Tanzania from July 2019 to June 2024. Data on socio-demographics, antiretroviral therapy (ART) status, CD4 count, treatment, and outcomes were extracted using a standardised data collection tool. The primary outcome was in-hospital survival (discharged alive vs died). Descriptive statistics summarised patient characteristics, and modified Poisson regression with robust variance estimated adjusted risk ratios (aRR) for factors associated with being discharged alive.

**Results:**

A total of 159 PHIV with CM records were reviewed. Of these, 89 (56.0%) were aged 36–55 years, 89 (56.0%) were female, and 138 (86.8%) were in WHO clinical stage IV. Of 159 patients, 88 (55.3%) received fluconazole monotherapy. In-hospital mortality among CM patients was 65 (46.5%). Discharge alive occurred in 61/73 (83.6%) of those on amphotericin B–based regimens versus 13/66 (19.7%) on fluconazole monotherapy. Patients treated with amphotericin B–based regimens were four times more likely to be discharged alive compared to those on fluconazole monotherapy (aPR = 4.19, 95% CI: 2.46–7.16, p < 0.001).

**Conclusion:**

CM remains a leading opportunistic infection causing high mortality among PHIV, with most patients managed using fluconazole monotherapy. In-hospital survival was significantly higher with amphotericin B–based regimens, highlighting the need to align practice with guideline recommendations. Further qualitative research is warranted to explore barriers to implementing recommended CM treatment.

## I. Introduction

Cryptococcus meningitis (CM) is the most common opportunistic infection and remains the leading cause of mortality in people with HIV (PHIV) [1]. Globally, CM is estimated to cause 350,000 to 1.5 million new cases annually, causing a mortality of approximately 181,000, with around 75% of deaths being reported in sub-Saharan Africa (SSA). In Africa prevalence of CM in PHIV ranges from 5.1% to 33%, with the majority of these cases and mortality occurring in SSA. For instance, studies conducted in South Africa, Ethiopia, Uganda, and Kenya reported a prevalence of 7%,7% 11%, and 33%Respectively [2,3]. Also, a study conducted in Dar es Salaam, Tanzania, reported a prevalence of 11.5% and an in-hospital mortality rate range of 50% to 70% [2,4–7].

Cryptococcal meningitis (CM) can be treated with various antifungal agents, including fluconazole, voriconazole, amphotericin B, flucytosine, and Posaconazole. However, susceptibility to these agents varies between *Cryptococcus gattii* and *Cryptococcus neoformans*, as well as across different global regions. Historically, CM treatment relied on fluconazole monotherapy at 800–1,200 mg daily doses. Over time, this approach has been associated with the emergence of antifungal resistance, limited fungicidal activity, and high mortality rates [8–11]. In response, the World Health Organisation (WHO) updated guidance to recommend amphotericin B–based induction, specifically single-dose liposomal amphotericin B (10 mg/kg) plus 14 days of flucytosine and fluconazole, which has demonstrated non-inferior survival, improved renal safety, and acceptable tolerability when appropriately monitored [12–14].

Amphotericin B-based regimen is reported to have more rapid clearance of *Cryptococcus* from both serum and cerebrospinal fluid (CSF) cultures. This regimen is highly fungicidal and significantly reduces in-hospital mortality compared to fluconazole monotherapy [10,11,14–16]. Guidelines recommend a single high dose (10 mg/kg) of liposomal amphotericin B, administered in combination with 14 days of flucytosine and fluconazole. This approach has demonstrated non-inferior survival outcomes, improved renal safety, and acceptable tolerability when patients are closely monitored [13].

Tanzania is a WHO member state with a significant burden of HIV [17,18]. Similar to many other SSA countries, the supply of antiretroviral and opportunistic infection (OI) medications in Tanzania is managed through a vertical program led by the government in collaboration with development partners [19,20]. PHIV access these medications free of charge through designated Care and Treatment Centres (CTCs), which are available in nearly all public healthcare facilities and a few private ones [21]. In alignment with WHO recommendations, Tanzania has updated its national HIV treatment guidelines to include the use of an amphotericin B–based regimen for the treatment of CM among PHIV. However, evidence on the implementation and clinical outcomes of this triple therapy regimen within routine care settings remains limited. Studies have reported several challenges affecting its uptake, including inconsistent supply of amphotericin B and flucytosine, limited healthcare provider skills in amphotericin B administration, concerns about drug toxicity, and inadequate laboratory infrastructure to support safe monitoring [22–24].

A recent study conducted in Tanzania reported that only 22.8% of 45 patients with CM received triple therapy [4]. However, the small sample size limited its ability to compare survival outcomes between patients treated with amphotericin B–based regimens and those receiving fluconazole monotherapy. To address this evidence gap, the present study examined, treatment modalities, outcomes, and factors associated with in-hospital survival among PHIV diagnosed with CM in two Tanzanian regional referral hospitals.

## II. Methods

### A. Study design and setting

This was a hospital-based, cross-sectional study involving retrospective data abstraction. Data were collected from patient records spanning July 2019 to June 2024. The abstraction process was conducted between March and April 2025 at Dodoma Regional Referral Hospital (DRRH) and Singida Regional Referral Hospital (SRRH) in Tanzania. Both facilities are secondary-level referral centres for the central zone, each hosting a CTC for PHIV and internal medicine wards providing inpatient care [25].

### B. Study population

The source population comprised all PHIV admitted to the medical wards of DRRH and SRRH within the study period. From this frame, we identified the CM cohort as those with a clinician diagnosis of CM documented in the chart and/or supporting laboratory evidence (e.g., positive CSF or serum cryptococcal antigen, India ink, or culture), recorded during the index admission.

### C. Sample size

The study included all available medical records of PHIV diagnosed with CM and admitted to the two selected regional referral hospitals from July 2019 to June 2024.

### D. Data collection procedure

Data were collected from patient files and electronic hospital management systems using a structured abstraction tool adapted from a previous study conducted in Dar es Salaam, Tanzania [4]. The tool was used to extract relevant information from patient medical records, including age, sex, marital status, health insurance status, occupation, antiretroviral therapy (ART) status, CD4 count, treatment modality, and treatment outcomes.

The data abstraction process was led by the principal investigator, a second-year Master’s student in the Hospital and Clinical Pharmacy program, in collaboration with two trained research assistants. Each assistant held a Bachelor of Pharmacy degree and was stationed at one of the selected RRHs. Before data collection, the research assistants were trained on the study objectives and received a comprehensive orientation on the data abstraction tool.

To ensure consistency and data quality, the principal investigator supervised the initial days of abstraction at each site and conducted ongoing consistency checks and logic/range checks to resolve discrepancies.

### E. Data analysis

Data was initially entered into Microsoft Excel and subsequently imported into IBM SPSS Statistics version 27 for cleaning and analysis. Categorical variables were summarised using frequencies and percentages. Bivariate associations with the primary outcome were screened using Pearson’s chi-square; variables with p ≤ 0.20 entered multivariable models alongside a priori confounders (site and calendar period). For the primary effect estimate, we fitted modified Poisson regression with robust variance to obtain adjusted risk ratios (aRRs) and 95% confidence intervals for being discharged alive comparing amphotericin B–based therapy versus fluconazole monotherapy. We assessed multicollinearity and retained variables with clinical relevance and/or statistical support. Mediator candidates (e.g., completion of induction) were not included in the primary model.

Twenty [20] CM patients who received treatment were excluded from this analysis, as their treatment outcomes could not be determined due to missing or incomplete records (i.e., loss to follow-up). Therefore, the regression analyses were conducted on the remaining patients with clearly documented outcomes, ensuring the validity of the associations identified.

### F. Ethical considerations

Ethical approval was obtained from the MUHAS Research and Ethics Committee (Ref. DA.282/298/01.C/2549). Hospital administrations at DRRH and SRRH granted permission for data access. No personal identifiers (e.g., names, hospital numbers) were abstracted. All procedures complied with institutional policies and the Declaration of Helsinki.

## III. Results

### A. Socio-demographic and clinical characteristics

A total of 159 patient medical records of PHIV with CM were reviewed. The majority of patients, 89 (56.0%), were aged between 36 and 55 years, and females accounted for 89 (56.0%). Most patients, 126 (79.4%), didn’t have active health insurance, and 116 (73.0%) were known to be HIV-positive before admission. Additionally, 138 (86.8%) were classified as WHO clinical stage IV. A history of ART default was recorded in 113 (71.1%). CD4 < 200 cells/mm3 was documented in 106 (66.7%) of the patients, and the majority 114 (71.7%) had at least one comorbidity, Table 1.

**Table 1:** Characteristics of participants in the study (N = 159).

Variable	Frequency	Percentage
**Year of Treatment**		
2019–2020	28	17.6%
2021–2022	44	27.7%
2023–2024	87	54.7%
**Sex**		
Female	89	56.0%
Male	70	44.0%
**Age in years (Mean: 43.94, Std: 12.28)**		
Below 35	42	26.4%
36–55	89	56.0%
Above 55	28	17.6%
**Insurance Status**		
Insured	33	20.6%
Not Ensured	126	79.4%
**Marital Status**		
Divorced	19	12.0%
Married	94	59.5%
Separated	8	5.1%
Single	23	14.6%
Widowed	14	8.9%
**Occupation**		
Employed	37	23.3%
Self Employed	77	48.4%
Student	8	5.0%
Unemployed	37	23.3%
**CD4 Count [Median: 182.81 (59.75–742.00)]**		
≥ 200 cells/mm3	53	33.3%
< 200 cells/mm3	106	66.7%
**HIV Diagnosis**		
Known HIV Patient at CM treatment	116	73.0%
Newly Diagnosed at CM treatment	43	27.0%
**WHO Clinical Stage**		
Stage I	4	2.5%
Stage II	8	5.0%
Stage III	9	5.7%
Stage IV	138	86.8%
**ART Defaulter**		
Yes	113	71.1%
No	46	28.9%
**Comorbidity**		
Yes	45	28.3%
No	114	71.7%
**Comorbidities (N = 45)** ^*^		
Chronic Kidney Disease	3	6.7%
Cardiovascular System disorder (Hypertension, Heart failure)	13	28.9%
Diabetes Mellitus Type 2	2	4.4%
Malignancy	2	4.4%
Opportunistic Infections (Toxoplasmosis, Pneumocystis Jirovecii pneumonia, pulmonary tuberculosis, Herpes)	25	55.6%

* One patient may have more than one comorbidity.

### B. Cryptococcal meningitis treatment modalities and outcomes

Of the 159 medical records of CM patients reviewed, 71 (44.7%) received an amphotericin B–based regimen. Baseline renal function tests were documented for 52 (73.2%) of these patients, and completion of the induction phase was reported for 48 (67.6%). In-hospital mortality was observed in 74 (46.5%) patients, Table 2.

**Table 2:** Cryptococcal meningitis treatment modalities, and outcome.

**Treatment Modality (N = 159)**		
Fluconazole Monotherapy	88	55.30%
Amphotericin B- based regimen	71	44.70%
**Completion of Induction Phase (N = 71)**		
Yes	48	67.60%
No	23	32.40%
**Renal Function Tests Done (N = 71)**		
Yes	52	73.20%
No	19	26.80%
**Treatment Outcomes (N = 139)**		
Discharged alive	65	40.9%
In-Hospital Mortality	74	46.5%
Lost to follow-up	19	11.9%
Referred	1	0.7%

### C. Factors associated with being discharged alive among study participants

In bivariable analyses, female sex and married/separated status were associated with higher crude likelihood of discharge alive; however, these associations were not statistically significant after adjustment. In the multivariable modified Poisson model, receipt of an amphotericin B–based regimen remained strongly associated with discharge alive compared with fluconazole monotherapy (aRR 4.19, 95% CI 2.46–7.16; p < 0.001), Table 3.

**Table 3:** Robust Poisson regression for factors associated with being discharged alive among participants (N = 139).

Variable				Univariate Analysis			Multivariable Analysis		
	In hospital mortality (%)	Discharged alive (%)	p – value	cPR	95% CI	p – value	aPR	95% CI	p – value
Sex									
Female	36(44.4)	45(55.6)	0.014	1.611	1.075-2.415	0.021	1.349	0.992-1.834	0.056
Male	38(65.5)	20(34.5)		1			1		
Age Groups									
< 35	21(60)	14(40)	0.379						
36-55	38(48.1)	41(51.9)							
> 55	15(60)	10(40)							
Insurance Status									
Insured	15(51.7)	14(48.3)	0.854						
Not Insured	59(53.6)	51(40.5)							
Marital Status									
Divorced	11(64.7)	6(35.3)	0.058	**1.765**	0.595-5.235	0.306	1.191	0.517-2.7422	0.682
Married	37(45.1)	45(54.9)		2.744	1.118-6.737	0.028	1.928	0.909-4.086	0.087
Separated	2(33.3)	4(66.7)		3.333	1.174-9.462	0.024	2.179	0.935-5.077	0.071
Widowed	8(57.1)	6(42.9)		2.143	0.739-6.216	0.161	1.379	0.611-3.112	0.439
Single	16(80)	4(20)		1			1		
Occupation									
Employed	17(53.1)	15(46.9)	0.354						
Student	6(85.7)	1(14.3)							
Self Employed	34(50)	34(50)							
Unemployed	17(53.2)	15(46.9)							
HIV Diagnosis									
Known HIV before CM Treatment	52(51.5)	49(48.5)	0.5	1.152	0.754-1.760	0.512			
Newly Diagnosed at CM Treatment	22(57.9)	16(46.8)		1					
WHO Clinical Stage									
Early stage (I and II)	3(37.5)	5(62.5)	0.358	1.365	0.773-2.409	0.284			
Advanced stage (III and IV)	71(54.2)	60(45.8)		1					
ART Defaulter									
Non-Defaulter	24(60)	16(40)	0.31	1.237	0.806-1.899	0.33			
Defaulter	50(50.5)	49(49.5)		1					
Comorbidity									
Without Comorbidity	54(55.1)	44(44.9)	0.496						
With Comorbidity	20(48.8)	21(51.2)							
CD4 Count Group									
< 200	53(55.8)	42(44.2)	0.002	2.046	1.223-3.422	0.006	1.299	0.857-1.967	0.217
> 200	12(27.3)	32(72.7)		1			1		
Treatment Modalities									
Amphotericin B Triple Therapy	12(16.4)	61(83.6)	<.0001	4.885	2.873-8.306	<0.001	4.192	2.456-7.155	<0.001
Fluconazole Monotherapy	53(80.3)	13(19.7)		1			1		

Key: cPR: crude Prevalence Ratio, aPR: adjusted Prevalence Ratio, Ref: Reference category.

## IV. Discussion

CM remains a major opportunistic infection among PHIV, and this study examined treatment modalities, outcomes, and factors associated with in-hospital survival among PHIV diagnosed with CM in Tanzania. A total of 159 medical records of PHIV diagnosed with CM were retrieved from July 2019 to June 2024. More than half of the patients (55.3%) were treated with fluconazole monotherapy. Among those who received an amphotericin B–based regimen, renal function tests were conducted for 73.2%, and 67.6% completed the induction phase. Overall, in-hospital mortality was 46.5%. Patients treated with amphotericin B–based regimens were four times more likely to be discharged alive compared to those who received fluconazole monotherapy.

This study demonstrates that CM continues to affect a considerable proportion of PHIV in regions where the disease is endemic. The magnitude observed in our cohort (159 CM patients) is comparable to that reported in Kenya (111 patients), but markedly higher than estimates from previous studies conducted in Dar es Salaam, Tanzania (48 patients), Ethiopia (16 and 14 patients), Cameroon (75 patients), and Sierra Leone (8 patients) [2,4,26–29]. These variations are likely attributable to differences in study design, population characteristics, and data sources; some were cross-sectional, prospective, or retrospective, while others focused on patients attending HIV clinics, hospital admissions, or data retrieved from medical records over time [2].

Although the scale-up of effective ART and the adoption of “test-and-treat” strategies have substantially reduced HIV-related morbidity, mortality, and transmission, CM remains a significant and life-threatening opportunistic infection. Our findings underscore the urgent need to strengthen early HIV diagnosis, timely ART initiation, and sustained adherence support, as well as to monitor emerging HIV-1 resistance patterns that may influence opportunistic infection risk. This is consistent with a multicentre study estimating an annual CM burden of approximately 162,500 cases in Africa, largely attributed to delayed ART initiation [6]. Supporting this, studies from Rwanda and South Africa reported a lower prevalence of CM, which was strongly associated with earlier ART initiation [30–32].

Our study revealed notable variability in CM treatment, with most patients receiving fluconazole monotherapy. This aligns with findings from a previous study in Dar es Salaam, Tanzania, where more than 80% of CM patients were treated with fluconazole monotherapy [4]. Such practice, however, contrasts with both national and WHO guidelines, which recommend amphotericin B–based regimens, preferably a combination of amphotericin B, flucytosine, and fluconazole, or amphotericin B with fluconazole when flucytosine is unavailable [14,33]. Process indicators revealed additional implementation gaps whereby baseline renal function testing was documented in 73.2% of amphotericin recipients, and only 67.6% completed the induction phase. Guideline recommendations emphasize renal monitoring due to the drug’s nephrotoxicity, and incomplete induction therapy has been strongly linked to poor outcomes [32]. Importantly, our study demonstrated that patients treated with amphotericin B-based regimens were four times more likely to be discharged alive compared to those receiving fluconazole monotherapy. These findings are consistent with evidence from multiple clinical trials showing superior effectiveness of amphotericin B-based therapy, reflected in shorter hospital stays and lower in-hospital mortality [7,12,34–36].

The continued reliance on fluconazole monotherapy and suboptimal completion of amphotericin B therapy likely stems from several factors, including limited awareness of treatment guidelines, inadequate provider training in amphotericin B preparation and administration, irregular drug supply, and the financial burden on patients and the facility. Addressing these systemic barriers is essential for improving CM management and reducing its persistently high mortality in resource-limited settings.

A key limitation of this study is the reliance on data abstracted from patients’ medical records, which was constrained by missing information and the inability to seek clarification from prescribers when needed. We mitigated these risks by using a piloted abstraction tool, training and on-site supervision of abstractors, drawing from both paper charts and electronic systems, and applying logic checks during cleaning. We prespecified covariates, adjusted for hospital site and calendar period, excluded patients with unknown disposition from the primary analysis, and ran sensitivity analyses to test robustness. Residual confounding remains possible, particularly from unmeasured severity markers including mental status, intracranial pressure, CrAg titer and lack of time-to-event data. Furthermore, as a purely quantitative study, it does not capture the perspectives and lived experiences of HCPs and patients regarding the burden of CM, treatment practices, outcomes, and the challenges and opportunities that could be leveraged to improve management of this life-threatening opportunistic infection among PHIV.

## V. Conclusion

This study demonstrates that CM remains a leading opportunistic infection causing high mortality among PHIV, with most patients still managed with fluconazole monotherapy despite national and international guideline recommendations. Patients treated with amphotericin B–based regimens had a markedly higher likelihood of survival at discharge, underscoring the urgent need to align clinical practice with evidence-based guidelines. We therefore recommend that CM in PHIV be managed using amphotericin B–based regimens as directed by local and WHO protocols. Additionally, future qualitative research should explore the barriers faced by HCPs, patients, and policymakers in implementing guideline-recommended CM treatment, to inform strategies that can strengthen CM management and reduce associated mortality.

## Supporting information

S1 File Analysis outputs generated using the Statistical Package for the Social Sciences (SPSS) software. (DOCX)

## Abbreviations

AIDS: acquired immunodeficiency syndrome
aPR: adjusted prevalence ratio
aRR: adjusted risk ratio
ART: antiretroviral therapy
CI: confidence interval
CM: cryptococcal meningitis
CrAg: cryptococcal antigen
cPR: crude prevalence ratio
CSF: cerebrospinal fluid
CTC: care and treatment centre
DRRH: Dodoma Regional Referral Hospital
HCPs: health care providers
HIV: human immunodeficiency virus
LTFU: lost to follow-up
MUHAS: Muhimbili University of Health and Allied Sciences
PHIV: people l with HIV
RRHs: regional referral hospitals
SSA: sub-Saharan Africa
SPSS: Statistical Package for the Social Sciences
SRRH: Singida Regional Referral Hospital
WHO: World Health Organization
