# Supplementary material for: In-hospital survival of adults with HIV-associated cryptococcal meningitis in Tanzania: A retrospective comparison of amphotericin B-based regimen and fluconazole monotherapy
Source: PLoS One. 2025 Dec 5;20(12):e0332786. doi: 10.1371/journal.pone.0332786 (PMC12680155; doi:10.1371/journal.pone.0332786)
Supplement: S1 File — (DOCX) [file pone.0332786.s001.docx]

**SPSS GENERATED ANALYSIS OUTPUT**

**Descriptives**

FREQUENCIES VARIABLES=treatyear_rec

/ORDER=ANALYSIS.

**Frequencies**

| **treatment year** | | | | | |
| --- | --- | --- | --- | --- | --- |
|  | | Frequency | Percent | Valid Percent | Cumulative Percent |
| Valid | 2019-2020 | 28 | 11.6 | 17.6 | 17.6 |
|  | 2021-2022 | 44 | 18.3 | 27.7 | 45.3 |
|  | 2023-2024 | 87 | 36.1 | 54.7 | 100.0 |
|  | Total | 159 | 66.0 | 100.0 |  |
| Missing | System | 82 | 34.0 |  |  |
| Total | | 241 | 100.0 |  |  |

FREQUENCIES VARIABLES=sex_rec

/ORDER=ANALYSIS.

**Frequencies**

| **sex new** | | | | | |
| --- | --- | --- | --- | --- | --- |
|  | | Frequency | Percent | Valid Percent | Cumulative Percent |
| Valid | Female | 89 | 36.9 | 56.0 | 56.0 |
|  | Male | 70 | 29.0 | 44.0 | 100.0 |
|  | Total | 159 | 66.0 | 100.0 |  |
| Missing | System | 82 | 34.0 |  |  |
| Total | | 241 | 100.0 |  |  |

FREQUENCIES VARIABLES=age_rec

/ORDER=ANALYSIS.

**Frequencies**

| **age groups** | | | | | |
| --- | --- | --- | --- | --- | --- |
|  | | Frequency | Percent | Valid Percent | Cumulative Percent |
| Valid | <35 | 42 | 17.4 | 26.4 | 26.4 |
|  | 36-55 | 89 | 36.9 | 56.0 | 82.4 |
|  | >55 | 28 | 11.6 | 17.6 | 100.0 |
|  | Total | 159 | 66.0 | 100.0 |  |
| Missing | System | 82 | 34.0 |  |  |
| Total | | 241 | 100.0 |  |  |

FREQUENCIES VARIABLES=marital_rec

/ORDER=ANALYSIS.

**Frequencies**

| **marital status** | | | | | |
| --- | --- | --- | --- | --- | --- |
|  | | Frequency | Percent | Valid Percent | Cumulative Percent |
| Valid | Divorced | 19 | 7.9 | 12.0 | 12.0 |
|  | Married | 94 | 39.0 | 59.5 | 71.5 |
|  | Separated | 8 | 3.3 | 5.1 | 76.6 |
|  | Single | 23 | 9.5 | 14.6 | 91.1 |
|  | Widowed | 14 | 5.8 | 8.9 | 100.0 |
|  | Total | 158 | 65.6 | 100.0 |  |
| Missing | System | 83 | 34.4 |  |  |
| Total | | 241 | 100.0 |  |  |

FREQUENCIES VARIABLES=occup_rec

/ORDER=ANALYSIS.

**Frequencies**

| **occupation** | | | | | |
| --- | --- | --- | --- | --- | --- |
|  | | Frequency | Percent | Valid Percent | Cumulative Percent |
| Valid | Employed | 37 | 15.4 | 23.3 | 23.3 |
|  | self employed | 77 | 32.0 | 48.4 | 71.7 |
|  | Student | 8 | 3.3 | 5.0 | 76.7 |
|  | Unemployed | 37 | 15.4 | 23.3 | 100.0 |
|  | Total | 159 | 66.0 | 100.0 |  |
| Missing | System | 82 | 34.0 |  |  |
| Total | | 241 | 100.0 |  |  |

FREQUENCIES VARIABLES=HIV_Diag_rec

/ORDER=ANALYSIS.

**Frequencies**

| **HIV Diagnosis** | | | | | |
| --- | --- | --- | --- | --- | --- |
|  | | Frequency | Percent | Valid Percent | Cumulative Percent |
| Valid | Newly Diagn | 43 | 17.8 | 27.0 | 27.0 |
|  | Known Diag | 116 | 48.1 | 73.0 | 100.0 |
|  | Total | 159 | 66.0 | 100.0 |  |
| Missing | System | 82 | 34.0 |  |  |
| Total | | 241 | 100.0 |  |  |

FREQUENCIES VARIABLES=WHO_Stage

/ORDER=ANALYSIS.

**Frequencies**

| **WHO Clinical stage** | | | | | |
| --- | --- | --- | --- | --- | --- |
|  | | Frequency | Percent | Valid Percent | Cumulative Percent |
| Valid | WHO Stage I | 4 | 1.7 | 2.5 | 2.5 |
|  | WHO Stage II | 8 | 3.3 | 5.0 | 7.5 |
|  | WHO Stage III | 9 | 3.7 | 5.7 | 13.2 |
|  | WHO Stage IV | 138 | 57.3 | 86.8 | 100.0 |
|  | Total | 159 | 66.0 | 100.0 |  |
| Missing | System | 82 | 34.0 |  |  |
| Total | | 241 | 100.0 |  |  |

FREQUENCIES VARIABLES=ART_rec

/ORDER=ANALYSIS.

**Frequencies**

| **ART Defaulter** | | | | | |
| --- | --- | --- | --- | --- | --- |
|  | | Frequency | Percent | Valid Percent | Cumulative Percent |
| Valid | Yes | 113 | 46.9 | 71.1 | 71.1 |
|  | No | 46 | 19.1 | 28.9 | 100.0 |
|  | Total | 159 | 66.0 | 100.0 |  |
| Missing | System | 82 | 34.0 |  |  |
| Total | | 241 | 100.0 |  |  |

FREQUENCIES VARIABLES=Comorb_rec

/ORDER=ANALYSIS.

**Frequencies**

| **Comorbidity** | | | | | |
| --- | --- | --- | --- | --- | --- |
|  | | Frequency | Percent | Valid Percent | Cumulative Percent |
| Valid | Yes | 114 | 47.3 | 71.7 | 71.7 |
|  | No | 45 | 18.7 | 28.3 | 100.0 |
|  | Total | 159 | 66.0 | 100.0 |  |
| Missing | System | 82 | 34.0 |  |  |
| Total | | 241 | 100.0 |  |  |

FREQUENCIES VARIABLES=CD4_rec

/ORDER=ANALYSIS.

**Frequencies**

| **Statistics** | | |
| --- | --- | --- |
| CD4 count | | |
| N | Valid | 159 |
|  | Missing | 82 |

| **CD4 count** | | | | | |
| --- | --- | --- | --- | --- | --- |
|  | | Frequency | Percent | Valid Percent | Cumulative Percent |
| Valid | <200 | 106 | 44.0 | 66.7 | 66.7 |
|  | >200 | 53 | 22.0 | 33.3 | 100.0 |
|  | Total | 159 | 66.0 | 100.0 |  |
| Missing | System | 82 | 34.0 |  |  |
| Total | | 241 | 100.0 |  |  |

FREQUENCIES VARIABLES=Treatment_Modalities

/ORDER=ANALYSIS.

**Frequencies**

| **Statistics** | | |
| --- | --- | --- |
| Treatment_Modalities | | |
| N | Valid | 241 |
|  | Missing | 0 |

| **Treatment_Modalities** | | | | | |
| --- | --- | --- | --- | --- | --- |
|  | | Frequency | Percent | Valid Percent | Cumulative Percent |
| Valid |  | 82 | 34.0 | 34.0 | 34.0 |
|  | Amphotericin B based-Triple therapy | 71 | 29.5 | 29.5 | 63.5 |
|  | Fluconazole monotherapy | 88 | 36.5 | 36.5 | 100.0 |
|  | Total | 241 | 100.0 | 100.0 |  |

FREQUENCIES VARIABLES=Comorb_TYPreco

/ORDER=ANALYSIS.

**Frequencies**

| **Statistics** | | |
| --- | --- | --- |
| Comorbidity type new | | |
| N | Valid | 241 |
|  | Missing | 0 |

DESCRIPTIVES VARIABLES=age_rec

/STATISTICS=MEAN STDDEV MIN MAX.

**Descriptives**

DESCRIPTIVES VARIABLES=Age

/STATISTICS=MEAN STDDEV MIN MAX.

**Descriptives**

| **Descriptive Statistics** | | | | | |
| --- | --- | --- | --- | --- | --- |
|  | N | Minimum | Maximum | Mean | Std. Deviation |
| Age | 159 | 16 | 78 | 43.94 | 12.278 |
| Valid N (listwise) | 159 |  |  |  |  |

FREQUENCIES VARIABLES=insur_rec

/ORDER=ANALYSIS.

**Frequencies**

| **Statistics** | | |
| --- | --- | --- |
| insurance status | | |
| N | Valid | 159 |
|  | Missing | 1 |

| **insurance status** | | | | | |
| --- | --- | --- | --- | --- | --- |
|  | | Frequency | Percent | Valid Percent | Cumulative Percent |
| Valid | Not insured | 126 | 78.8 | 79.2 | 79.2 |
|  | Insured | 33 | 20.6 | 20.8 | 100.0 |
|  | Total | 159 | 99.4 | 100.0 |  |
| Missing | System | 1 | .6 |  |  |
| Total | | 160 | 100.0 |  |  |

FREQUENCIES VARIABLES=Comorb_TYPreco

/ORDER=ANALYSIS.

| **Comorbidity** | | | | | |
| --- | --- | --- | --- | --- | --- |
|  | | Frequency | Percent | Valid Percent | Cumulative Percent |
| Valid | No | 114 | 71.3 | 71.7 | 71.7 |
|  | Yes | 45 | 28.1 | 28.3 | 100.0 |
|  | Total | 159 | 99.4 | 100.0 |  |
| Missing | System | 1 | .6 |  |  |
| Total | | 160 | 100.0 |  |  |

| **comorbidity types** | | | | | |
| --- | --- | --- | --- | --- | --- |
|  | | Frequency | Percent | Valid Percent | Cumulative Percent |
| Valid | Malignancy | 2 | 1.3 | 4.4 | 4.7 |
|  | Chronic Kidney Disease | 3 | 1.9 | 6.7 | 11.6 |
|  | Cardiovascular System disorder (Hypertension, Heart failure) | 13 | 8.1 | 28.9 | 41.9 |
|  | Opportunistic Infections (Toxoplasmosis, Pneumocystis Jirovecii pneumonia, pulmonary tuberculosis, Herpes) | 25 | 15.6 | 55.6 | 100.0 |
|  | Diabetes Mellitus Type 2  Total | 2  45 | 1.3  28.2 | 4.4  100.0 |  |
| Missing | System | 114 | 71.8 |  |  |
| Total | | 160 | 100.0 |  |  |

| **Diagnosis_Investigation** | | | | | |
| --- | --- | --- | --- | --- | --- |
|  | | Frequency | Percent | Valid Percent | Cumulative Percent |
| Valid |  | 81 | 12.6 | 12.6 | 12.6 |
|  | Clinical Presentation | 16 | 2.5 | 2.5 | 15.1 |
|  | Clinical Presentation, Serum or CSF Cryptococcus antigen (CrAg) test | 6 | .9 | .9 | 16.0 |
|  | CSF cultures | 1 | .2 | .2 | 16.2 |
|  | CSF cultures, Serum or CSF Cryptococcus antigen (CrAg) test | 2 | .3 | .3 | 16.5 |
|  | CSF gram stain, Serum or CSF Cryptococcus antigen (CrAg) test | 14 | 2.2 | 2.2 | 18.7 |
|  | CSF gram stain, CSF India Ink stain test, , Clinical Presentation | 1 | .2 | .2 | 18.8 |
|  | CSF gram stain, CSF India Ink stain test, Clinical Presentation | 2 | .3 | .3 | 19.2 |
|  | CSF gram stain, CSF India Ink stain test, Serum or CSF Cryptococcus antigen (CrAg) test | 2 | .3 | .3 | 19.5 |
|  | CSF gram stain, Serum or CSF Cryptococcus antigen (CrAg) test | 3 | .5 | .5 | 19.9 |
|  | CSF gram stain, Serum or CSF Cryptococcus antigen (CrAg) test, Clinical Presentation | 7 | 1.1 | 1.1 | 21.0 |
|  | Serum or CSF Cryptococcus antigen (CrAg) test | 477 | 74.3 | 74.3 | 95.3 |
|  | Serum or CSF Cryptococcus antigen (CrAg) test, | 1 | .2 | .2 | 95.5 |
|  | Serum or CSF Cryptococcus antigen (CrAg) test, , Clinical Presentation | 1 | .2 | .2 | 95.6 |
|  | Serum or CSF Cryptococcus antigen (CrAg) test, Clinical Presentation | 23 | 3.6 | 3.6 | 99.2 |
|  | Serum or CSF Cryptococcus antigen (CrAg) test, CSF cultures | 2 | .3 | .3 | 99.5 |
|  | Serum or CSF Cryptococcus antigen (CrAg) test, CSF cultures, CSF gram stain | 1 | .2 | .2 | 99.7 |
|  | Serum or CSF Cryptococcus antigen (CrAg) test, CSF gram stain, CSF cultures | 2 | .3 | .3 | 100.0 |
|  | Total | 642 | 100.0 | 100.0 |  |

| **Treatment_Modalities** | | | | | |
| --- | --- | --- | --- | --- | --- |
|  | | Frequency | Percent | Valid Percent | Cumulative Percent |
| Valid |  | 483 | 75.2 | 75.2 | 75.2 |
|  | Amphotericin B based-Triple therapy | 71 | 11.1 | 11.1 | 86.3 |
|  | Fluconazole monotherapy | 88 | 13.7 | 13.7 | 100.0 |
|  | Total | 642 | 100.0 | 100.0 |  |

| **Completed_induction** | | | | | |
| --- | --- | --- | --- | --- | --- |
|  | | Frequency | Percent | Valid Percent | Cumulative Percent |
| Valid |  | 571 | 88.9 | 88.9 | 88.9 |
|  | No | 23 | 3.6 | 3.6 | 92.5 |
|  | Yes | 48 | 7.5 | 7.5 | 100.0 |
|  | Total | 642 | 100.0 | 100.0 |  |

| **Outcome** | | | | | |
| --- | --- | --- | --- | --- | --- |
|  | | Frequency | Percent | Valid Percent | Cumulative Percent |
| Valid |  | 482 | 75.1 | 75.1 | 75.1 |
|  | Discharged Alive | 66 | 10.3 | 10.3 | 85.4 |
|  | In-Hospital Mortality | 74 | 11.5 | 11.5 | 96.9 |
|  | Lost to follow up | 19 | 3.0 | 3.0 | 99.8 |
|  | Referred | 1 | .2 | .2 | 100.0 |
|  | Total | 642 | 100.0 | 100.0 |  |

| **RFT_Done** | | | | | |
| --- | --- | --- | --- | --- | --- |
|  | | Frequency | Percent | Valid Percent | Cumulative Percent |
| Valid |  | 571 | 88.9 | 88.9 | 88.9 |
|  | No | 19 | 3.0 | 3.0 | 91.9 |
|  | Yes | 52 | 8.1 | 8.1 | 100.0 |
|  | Total | 642 | 100.0 | 100.0 |  |

***Table 3: Robust Poisson regression for factors associated with being discharged alive among participants (N = 139)***

**Age (Binned) * Outcome_recorded**

| **Crosstab** | | | | | |
| --- | --- | --- | --- | --- | --- |
|  | | | Outcome_recorded | | Total |
|  |  |  | In hospital mortality | Discharge Alive |  |
| Age (Binned) | <= 35 | Count | 21 | 14 | 35 |
|  |  | % within Age (Binned) | 60.0% | 40.0% | 100.0% |
|  | 36 - 55 | Count | 38 | 41 | 79 |
|  |  | % within Age (Binned) | 48.1% | 51.9% | 100.0% |
|  | > 55 | Count | 15 | 10 | 25 |
|  |  | % within Age (Binned) | 60.0% | 40.0% | 100.0% |
| Total | | Count | 74 | 65 | 139 |
|  |  | % within Age (Binned) | 53.2% | 46.8% | 100.0% |

| **Chi-Square Tests** | | | | | | |
| --- | --- | --- | --- | --- | --- | --- |
|  | Value | df | Asymptotic Significance (2-sided) | Exact Sig. (2-sided) | Exact Sig. (1-sided) | Point Probability |
| Pearson Chi-Square | 1.939^a^ | 2 | .379 | .405 |  |  |
| Likelihood Ratio | 1.947 | 2 | .378 | .405 |  |  |
| Fisher-Freeman-Halton Exact Test | 1.915 |  |  | .405 |  |  |
| Linear-by-Linear Association | .031^b^ | 1 | .861 | .897 | .482 | .101 |
| N of Valid Cases | 139 |  |  |  |  |  |
| a. 0 cells (.0%) have expected count less than 5. The minimum expected count is 11.69. | | | | | | |
| b. The standardized statistic is .175. | | | | | | |

**Sex_recorded * Outcome_recorded**

| **Crosstab** | | | | | |
| --- | --- | --- | --- | --- | --- |
|  | | | Outcome_recorded | | Total |
|  |  |  | In hospital mortality | Discharge Alive |  |
| Sex_recorded | Female | Count | 36 | 45 | 81 |
|  |  | % within Sex_recorded | 44.4% | 55.6% | 100.0% |
|  | Male | Count | 38 | 20 | 58 |
|  |  | % within Sex_recorded | 65.5% | 34.5% | 100.0% |
| Total | | Count | 74 | 65 | 139 |
|  |  | % within Sex_recorded | 53.2% | 46.8% | 100.0% |

| **Chi-Square Tests** | | | | | | |
| --- | --- | --- | --- | --- | --- | --- |
|  | Value | df | Asymptotic Significance (2-sided) | Exact Sig. (2-sided) | Exact Sig. (1-sided) | Point Probability |
| Pearson Chi-Square | 6.029^a^ | 1 | .014 | .016 | .011 |  |
| Continuity Correction^b^ | 5.212 | 1 | .022 |  |  |  |
| Likelihood Ratio | 6.098 | 1 | .014 | .016 | .011 |  |
| Fisher's Exact Test |  |  |  | .016 | .011 |  |
| Linear-by-Linear Association | 5.985^c^ | 1 | .014 | .016 | .011 | .007 |
| N of Valid Cases | 139 |  |  |  |  |  |
| a. 0 cells (.0%) have expected count less than 5. The minimum expected count is 27.12. | | | | | | |
| b. Computed only for a 2x2 table | | | | | | |
| c. The standardized statistic is -2.447. | | | | | | |

**treatment_modalities2 * Outcome_recorded**

| **Crosstab** | | | | | |
| --- | --- | --- | --- | --- | --- |
|  | | | Outcome_recorded | | Total |
|  |  |  | In hospital mortality | Discharge Alive |  |
| treatment_modalities2 | Amphotericin B based-Triple therapy | Count | 13 | 53 | 66 |
|  |  | % within treatment_modalities2 | 19.7% | 80.3% | 100.0% |
|  | Fluconazole monotherapy | Count | 61 | 12 | 73 |
|  |  | % within treatment_modalities2 | 83.6% | 16.4% | 100.0% |
| Total | | Count | 74 | 65 | 139 |
|  |  | % within treatment_modalities2 | 53.2% | 46.8% | 100.0% |

| **Chi-Square Tests** | | | | | | |
| --- | --- | --- | --- | --- | --- | --- |
|  | Value | df | Asymptotic Significance (2-sided) | Exact Sig. (2-sided) | Exact Sig. (1-sided) | Point Probability |
| Pearson Chi-Square | 56.788^a^ | 1 | .000 | .000 | .000 |  |
| Continuity Correction^b^ | 54.252 | 1 | .000 |  |  |  |
| Likelihood Ratio | 61.374 | 1 | .000 | .000 | .000 |  |
| Fisher's Exact Test |  |  |  | .000 | .000 |  |
| Linear-by-Linear Association | 56.380^c^ | 1 | .000 | .000 | .000 | .000 |
| N of Valid Cases | 139 |  |  |  |  |  |
| a. 0 cells (.0%) have expected count less than 5. The minimum expected count is 30.86. | | | | | | |
| b. Computed only for a 2x2 table | | | | | | |
| c. The standardized statistic is -7.509. | | | | | | |

**Insurance_status_recorded * Outcome_recorded**

| **Crosstab** | | | | | |
| --- | --- | --- | --- | --- | --- |
|  | | | Outcome_recorded | | Total |
|  |  |  | In hospital mortality | Discharge Alive |  |
| Insurance_status_recorded | Insured | Count | 15 | 14 | 29 |
|  |  | % within Insurance_status_recorded | 51.7% | 48.3% | 100.0% |
|  | Not Ensured | Count | 59 | 51 | 110 |
|  |  | % within Insurance_status_recorded | 53.6% | 46.4% | 100.0% |
| Total | | Count | 74 | 65 | 139 |
|  |  | % within Insurance_status_recorded | 53.2% | 46.8% | 100.0% |

| **Chi-Square Tests** | | | | | | |
| --- | --- | --- | --- | --- | --- | --- |
|  | Value | df | Asymptotic Significance (2-sided) | Exact Sig. (2-sided) | Exact Sig. (1-sided) | Point Probability |
| Pearson Chi-Square | .034^a^ | 1 | .854 | 1.000 | .509 |  |
| Continuity Correction^b^ | .000 | 1 | 1.000 |  |  |  |
| Likelihood Ratio | .034 | 1 | .854 | 1.000 | .509 |  |
| Fisher's Exact Test |  |  |  | 1.000 | .509 |  |
| Linear-by-Linear Association | .033^c^ | 1 | .855 | 1.000 | .509 | .162 |
| N of Valid Cases | 139 |  |  |  |  |  |
| a. 0 cells (.0%) have expected count less than 5. The minimum expected count is 13.56. | | | | | | |
| b. Computed only for a 2x2 table | | | | | | |
| c. The standardized statistic is -.183. | | | | | | |

**HIV_diagnosis_recorded * Outcome_recorded**

| **Crosstab** | | | | | |
| --- | --- | --- | --- | --- | --- |
|  | | | Outcome_recorded | | Total |
|  |  |  | In hospital mortality | Discharge Alive |  |
| HIV_diagnosis_recorded | Known HIV patient | Count | 52 | 49 | 101 |
|  |  | % within HIV_diagnosis_recorded | 51.5% | 48.5% | 100.0% |
|  | Newly diagnosed | Count | 22 | 16 | 38 |
|  |  | % within HIV_diagnosis_recorded | 57.9% | 42.1% | 100.0% |
| Total | | Count | 74 | 65 | 139 |
|  |  | % within HIV_diagnosis_recorded | 53.2% | 46.8% | 100.0% |

| **Chi-Square Tests** | | | | | | |
| --- | --- | --- | --- | --- | --- | --- |
|  | Value | df | Asymptotic Significance (2-sided) | Exact Sig. (2-sided) | Exact Sig. (1-sided) | Point Probability |
| Pearson Chi-Square | .456^a^ | 1 | .500 | .569 | .315 |  |
| Continuity Correction^b^ | .235 | 1 | .628 |  |  |  |
| Likelihood Ratio | .457 | 1 | .499 | .569 | .315 |  |
| Fisher's Exact Test |  |  |  | .569 | .315 |  |
| Linear-by-Linear Association | .452^c^ | 1 | .501 | .569 | .315 | .121 |
| N of Valid Cases | 139 |  |  |  |  |  |
| a. 0 cells (.0%) have expected count less than 5. The minimum expected count is 17.77. | | | | | | |
| b. Computed only for a 2x2 table | | | | | | |
| c. The standardized statistic is -.673. | | | | | | |

**Marital_status_recorded * Outcome_recorded**

| **Crosstab** | | | | | |
| --- | --- | --- | --- | --- | --- |
|  | | | Outcome_recorded | | Total |
|  |  |  | In hospital mortality | Discharge Alive |  |
| Marital_status_recorded | Divorced | Count | 11 | 6 | 17 |
|  |  | % within Marital_status_recorded | 64.7% | 35.3% | 100.0% |
|  | Married | Count | 37 | 45 | 82 |
|  |  | % within Marital_status_recorded | 45.1% | 54.9% | 100.0% |
|  | Separated | Count | 2 | 4 | 6 |
|  |  | % within Marital_status_recorded | 33.3% | 66.7% | 100.0% |
|  | Single | Count | 16 | 4 | 20 |
|  |  | % within Marital_status_recorded | 80.0% | 20.0% | 100.0% |
|  | Widowed | Count | 8 | 6 | 14 |
|  |  | % within Marital_status_recorded | 57.1% | 42.9% | 100.0% |
| Total | | Count | 74 | 65 | 139 |
|  |  | % within Marital_status_recorded | 53.2% | 46.8% | 100.0% |

| **Chi-Square Tests** | | | | | | |
| --- | --- | --- | --- | --- | --- | --- |
|  | Value | df | Asymptotic Significance (2-sided) | Exact Sig. (2-sided) | Exact Sig. (1-sided) | Point Probability |
| Pearson Chi-Square | 9.862^a^ | 4 | .043 | .040 |  |  |
| Likelihood Ratio | 10.367 | 4 | .035 | .049 |  |  |
| Fisher-Freeman-Halton Exact Test | 9.916 |  |  | .038 |  |  |
| Linear-by-Linear Association | 1.752^b^ | 1 | .186 | .197 | .106 | .024 |
| N of Valid Cases | 139 |  |  |  |  |  |
| a. 2 cells (20.0%) have expected count less than 5. The minimum expected count is 2.81. | | | | | | |
| b. The standardized statistic is -1.324. | | | | | | |

**Occupation_recorded * Outcome_recorded**

| **Crosstab** | | | | | |
| --- | --- | --- | --- | --- | --- |
|  | | | Outcome_recorded | | Total |
|  |  |  | In hospital mortality | Discharge Alive |  |
| Occupation_recorded | Employed | Count | 17 | 15 | 32 |
|  |  | % within Occupation_recorded | 53.1% | 46.9% | 100.0% |
|  | Self employed | Count | 34 | 34 | 68 |
|  |  | % within Occupation_recorded | 50.0% | 50.0% | 100.0% |
|  | Student | Count | 6 | 1 | 7 |
|  |  | % within Occupation_recorded | 85.7% | 14.3% | 100.0% |
|  | Unemployed | Count | 17 | 15 | 32 |
|  |  | % within Occupation_recorded | 53.1% | 46.9% | 100.0% |
| Total | | Count | 74 | 65 | 139 |
|  |  | % within Occupation_recorded | 53.2% | 46.8% | 100.0% |

| **Chi-Square Tests** | | | | | | |
| --- | --- | --- | --- | --- | --- | --- |
|  | Value | df | Asymptotic Significance (2-sided) | Exact Sig. (2-sided) | Exact Sig. (1-sided) | Point Probability |
| Pearson Chi-Square | 3.252^a^ | 3 | .354 | .363 |  |  |
| Likelihood Ratio | 3.629 | 3 | .304 | .322 |  |  |
| Fisher-Freeman-Halton Exact Test | 3.126 |  |  | .377 |  |  |
| Linear-by-Linear Association | .128^b^ | 1 | .721 | .750 | .391 | .060 |
| N of Valid Cases | 139 |  |  |  |  |  |
| a. 2 cells (25.0%) have expected count less than 5. The minimum expected count is 3.27. | | | | | | |
| b. The standardized statistic is -.358. | | | | | | |

**WHO_clinical_stage_recorded * Outcome_recorded**

| **Crosstab** |  |  |  |  |  |
| --- | --- | --- | --- | --- | --- |
|  | Outcome_recorded | Total |  |  |  |
|  | In hospital mortality | Discharge Alive |  |  |  |
| WHO_clinical_stage_recorded | Early stage (I & II) | Count | 3 | 5 | 8 |
|  |  | % within WHO_clinical_stage_recorded | 37.5% | 62.5% | 100.0% |
|  | Advanced stage (III & IV) | Count | 71 | 60 | 131 |
|  |  | % within WHO_clinical_stage_recorded | 54.2% | 45.8% | 100.0% |
| Total | Count | 74 | 65 | 139 |  |
|  | % within WHO_clinical_stage_recorded | 53.2% | 46.8% | 100.0% |  |

| **Chi-Square Tests** | | | | | | |
| --- | --- | --- | --- | --- | --- | --- |
|  | Value | df | Asymptotic Significance (2-sided) | Exact Sig. (2-sided) | Exact Sig. (1-sided) | Point Probability |
| Pearson Chi-Square | .844^a^ | 1 | .358 | .473 | .290 |  |
| Continuity Correction^b^ | .307 | 1 | .580 |  |  |  |
| Likelihood Ratio | .847 | 1 | .357 | .473 | .290 |  |
| Fisher's Exact Test |  |  |  | .473 | .290 |  |
| Linear-by-Linear Association | .838^c^ | 1 | .360 | .473 | .290 | .190 |
| N of Valid Cases | 139 |  |  |  |  |  |
| a. 2 cells (50.0%) have expected count less than 5. The minimum expected count is 3.74. | | | | | | |
| b. Computed only for a 2x2 table | | | | | | |
| c. The standardized statistic is -.916. | | | | | | |

**ART_Defaulter_recorded * Outcome_recorded**

| **Crosstab** | | | | | |
| --- | --- | --- | --- | --- | --- |
|  | | | Outcome_recorded | | Total |
|  |  |  | In hospital mortality | Discharge Alive |  |
| ART_Defaulter_recorded | No | Count | 24 | 16 | 40 |
|  |  | % within ART_Defaulter_recorded | 60.0% | 40.0% | 100.0% |
|  | Yes | Count | 50 | 49 | 99 |
|  |  | % within ART_Defaulter_recorded | 50.5% | 49.5% | 100.0% |
| Total | | Count | 74 | 65 | 139 |
|  |  | % within ART_Defaulter_recorded | 53.2% | 46.8% | 100.0% |

| **Chi-Square Tests** | | | | | | |
| --- | --- | --- | --- | --- | --- | --- |
|  | Value | df | Asymptotic Significance (2-sided) | Exact Sig. (2-sided) | Exact Sig. (1-sided) | Point Probability |
| Pearson Chi-Square | 1.032^a^ | 1 | .310 | .351 | .204 |  |
| Continuity Correction^b^ | .686 | 1 | .408 |  |  |  |
| Likelihood Ratio | 1.038 | 1 | .308 | .351 | .204 |  |
| Fisher's Exact Test |  |  |  | .351 | .204 |  |
| Linear-by-Linear Association | 1.024^c^ | 1 | .312 | .351 | .204 | .090 |
| N of Valid Cases | 139 |  |  |  |  |  |
| a. 0 cells (.0%) have expected count less than 5. The minimum expected count is 18.71. | | | | | | |
| b. Computed only for a 2x2 table | | | | | | |
| c. The standardized statistic is 1.012. | | | | | | |

**Comorbidity_recorded * Outcome_recorded**

| **Crosstab** | | | | | |
| --- | --- | --- | --- | --- | --- |
|  | | | Outcome_recorded | | Total |
|  |  |  | In hospital mortality | Discharge Alive |  |
| Comorbidity_recorded | No | Count | 54 | 44 | 98 |
|  |  | % within Comorbidity_recorded | 55.1% | 44.9% | 100.0% |
|  | Yes | Count | 20 | 21 | 41 |
|  |  | % within Comorbidity_recorded | 48.8% | 51.2% | 100.0% |
| Total | | Count | 74 | 65 | 139 |
|  |  | % within Comorbidity_recorded | 53.2% | 46.8% | 100.0% |

| **Chi-Square Tests** | | | | | | |
| --- | --- | --- | --- | --- | --- | --- |
|  | Value | df | Asymptotic Significance (2-sided) | Exact Sig. (2-sided) | Exact Sig. (1-sided) | Point Probability |
| Pearson Chi-Square | .464^a^ | 1 | .496 | .577 | .310 |  |
| Continuity Correction^b^ | .245 | 1 | .621 |  |  |  |
| Likelihood Ratio | .463 | 1 | .496 | .577 | .310 |  |
| Fisher's Exact Test |  |  |  | .577 | .310 |  |
| Linear-by-Linear Association | .461^c^ | 1 | .497 | .577 | .310 | .117 |
| N of Valid Cases | 139 |  |  |  |  |  |
| a. 0 cells (.0%) have expected count less than 5. The minimum expected count is 19.17. | | | | | | |
| b. Computed only for a 2x2 table | | | | | | |
| c. The standardized statistic is .679. | | | | | | |

**Completion_induction_recorded * Outcome_recorded**

| **Crosstab** | | | | | |
| --- | --- | --- | --- | --- | --- |
|  | | | Outcome_recorded | | Total |
|  |  |  | In hospital mortality | Discharge Alive |  |
| Completion_induction_recorded | No | Count | 11 | 7 | 18 |
|  |  | % within Completion_induction_recorded | 61.1% | 38.9% | 100.0% |
|  | Yes | Count | 2 | 46 | 48 |
|  |  | % within Completion_induction_recorded | 4.2% | 95.8% | 100.0% |
| Total | | Count | 13 | 53 | 66 |
|  |  | % within Completion_induction_recorded | 19.7% | 80.3% | 100.0% |

| **Chi-Square Tests** | | | | | | |
| --- | --- | --- | --- | --- | --- | --- |
|  | Value | df | Asymptotic Significance (2-sided) | Exact Sig. (2-sided) | Exact Sig. (1-sided) | Point Probability |
| Pearson Chi-Square | 26.837^a^ | 1 | .000 | .000 | .000 |  |
| Continuity Correction^b^ | 23.358 | 1 | .000 |  |  |  |
| Likelihood Ratio | 24.810 | 1 | .000 | .000 | .000 |  |
| Fisher's Exact Test |  |  |  | .000 | .000 |  |
| Linear-by-Linear Association | 26.431^c^ | 1 | .000 | .000 | .000 | .000 |
| N of Valid Cases | 66 |  |  |  |  |  |
| a. 1 cells (25.0%) have expected count less than 5. The minimum expected count is 3.55. | | | | | | |
| b. Computed only for a 2x2 table | | | | | | |
| c. The standardized statistic is 5.141. | | | | | | |

**RFT_Done_recorded * Outcome_recorded**

| **Crosstab** | | | | | |
| --- | --- | --- | --- | --- | --- |
|  | | | Outcome_recorded | | Total |
|  |  |  | In hospital mortality | Discharge Alive |  |
| RFT_Done_recorded | No | Count | 6 | 9 | 15 |
|  |  | % within RFT_Done_recorded | 40.0% | 60.0% | 100.0% |
|  | Yes | Count | 7 | 44 | 51 |
|  |  | % within RFT_Done_recorded | 13.7% | 86.3% | 100.0% |
| Total | | Count | 13 | 53 | 66 |
|  |  | % within RFT_Done_recorded | 19.7% | 80.3% | 100.0% |

| **Chi-Square Tests** | | | | | | |
| --- | --- | --- | --- | --- | --- | --- |
|  | Value | df | Asymptotic Significance (2-sided) | Exact Sig. (2-sided) | Exact Sig. (1-sided) | Point Probability |
| Pearson Chi-Square | 5.059^a^ | 1 | .024 | .035 | .035 |  |
| Continuity Correction^b^ | 3.534 | 1 | .060 |  |  |  |
| Likelihood Ratio | 4.510 | 1 | .034 | .058 | .035 |  |
| Fisher's Exact Test |  |  |  | .058 | .035 |  |
| Linear-by-Linear Association | 4.982^c^ | 1 | .026 | .035 | .035 | .028 |
| N of Valid Cases | 66 |  |  |  |  |  |
| a. 1 cells (25.0%) have expected count less than 5. The minimum expected count is 2.95. | | | | | | |
| b. Computed only for a 2x2 table | | | | | | |
| c. The standardized statistic is 2.232. | | | | | | |

| **CD4_Count (Binned) * Outcome_recorded Crosstabulation** | | | | | |
| --- | --- | --- | --- | --- | --- |
|  | | | Outcome_recorded | | Total |
|  |  |  | In hospital mortality | Discharge Alive |  |
| CD4_Count (Binned) | < 200.0 | Count | 42 | 53 | 95 |
|  |  | % within CD4_Count (Binned) | 44.2% | 55.8% | 100.0% |
|  | > = 200.0 | Count | 32 | 12 | 44 |
|  |  | % within CD4_Count (Binned) | 72.7% | 27.3% | 100.0% |
| Total | | Count | 74 | 65 | 139 |
|  |  | % within CD4_Count (Binned) | 53.2% | 46.8% | 100.0% |

| **Chi-Square Tests** | | | | | | |
| --- | --- | --- | --- | --- | --- | --- |
|  | Value | df | Asymptotic Significance (2-sided) | Exact Sig. (2-sided) | Exact Sig. (1-sided) | Point Probability |
| Pearson Chi-Square | 9.823^a^ | 1 | .002 | .002 | .001 |  |
| Continuity Correction^b^ | 8.711 | 1 | .003 |  |  |  |
| Likelihood Ratio | 10.127 | 1 | .001 | .002 | .001 |  |
| Fisher's Exact Test |  |  |  | .002 | .001 |  |
| Linear-by-Linear Association | 9.752^c^ | 1 | .002 | .002 | .001 | .001 |
| N of Valid Cases | 139 |  |  |  |  |  |
| a. 0 cells (.0%) have expected count less than 5. The minimum expected count is 20.58. | | | | | | |
| b. Computed only for a 2x2 table | | | | | | |
| c. The standardized statistic is -3.123. | | | | | | |

**UNIVARIATE AND MULTIVARIABLE**

| **Categorical Variable Information** | | | | |
| --- | --- | --- | --- | --- |
|  | | | N | Percent |
| Factor | Sex_recorded | Female | 81 | 58.3% |
|  |  | Male | 58 | 41.7% |
|  |  | Total | 139 | 100.0% |

| **Omnibus Test^a^** | | |
| --- | --- | --- |
| Likelihood Ratio Chi-Square | df | Sig. |
| 3.322 | 1 | .068 |
| Dependent Variable: Outcome_recorded  Model: (Intercept), Sex_recorded^a^ | | |
| a. Compares the fitted model against the intercept-only model. | | |

| **Tests of Model Effects** | | | |
| --- | --- | --- | --- |
| Source | Type III | | |
|  | Wald Chi-Square | df | Sig. |
| (Intercept) | 64.049 | 1 | .000 |
| Sex_recorded | 5.335 | 1 | .021 |
| Dependent Variable: Outcome_recorded  Model: (Intercept), Sex_recorded | | | |

| **Parameter Estimates** | | | | | | | | | | |
| --- | --- | --- | --- | --- | --- | --- | --- | --- | --- | --- |
| Parameter | B | Std. Error | 95% Wald Confidence Interval | | Hypothesis Test | | | Exp(B) | 95% Wald Confidence Interval for Exp(B) | |
|  |  |  | Lower | Upper | Wald Chi-Square | df | Sig. |  | Lower | Upper |
| (Intercept) | -1.065 | .1810 | -1.419 | -.710 | 34.605 | 1 | .000 | .345 | .242 | .492 |
| [Sex_recorded=1] | .477 | .2065 | .072 | .882 | 5.335 | 1 | .021 | 1.611 | 1.075 | 2.415 |
| [Sex_recorded=2] | 0^a^ | . | . | . | . | . | . | 1 | . | . |
| (Scale) | 1^b^ |  |  |  |  |  |  |  |  |  |
| Dependent Variable: Outcome_recorded  Model: (Intercept), Sex_recorded | | | | | | | | | | |
| a. Set to zero because this parameter is redundant. | | | | | | | | | | |
| b. Fixed at the displayed value. | | | | | | | | | | |

**treatment_modalities2**

| **Categorical Variable Information** | | | | |
| --- | --- | --- | --- | --- |
|  | | | N | Percent |
| Factor | treatment_modalities2 | Amphotericin B based-Triple therapy | 66 | 47.5% |
|  |  | Fluconazole monotherapy | 73 | 52.5% |
|  |  | Total | 139 | 100.0% |

| **Goodness of Fit^a^** | | | |
| --- | --- | --- | --- |
|  | Value | df | Value/df |
| Deviance | 66.586 | 137 | .486 |
| Scaled Deviance | 66.586 | 137 |  |
| Pearson Chi-Square | 74.000 | 137 | .540 |
| Scaled Pearson Chi-Square | 74.000 | 137 |  |
| Log Likelihood^b^ | -98.293 |  |  |
| Akaike's Information Criterion (AIC) | 200.586 |  |  |
| Finite Sample Corrected AIC (AICC) | 200.674 |  |  |
| Bayesian Information Criterion (BIC) | 206.455 |  |  |
| Consistent AIC (CAIC) | 208.455 |  |  |
| Dependent Variable: Outcome_recorded  Model: (Intercept), treatment_modalities2^a^ | | | |
| a. Information criteria are in smaller-is-better form. | | | |
| b. The full log likelihood function is displayed and used in computing information criteria. | | | |

| **Omnibus Test^a^** | | |
| --- | --- | --- |
| Likelihood Ratio Chi-Square | df | Sig. |
| 32.226 | 1 | .000 |
| Dependent Variable: Outcome_recorded  Model: (Intercept), treatment_modalities2^a^ | | |
| a. Compares the fitted model against the intercept-only model. | | |

| **Tests of Model Effects** | | | |
| --- | --- | --- | --- |
| Source | Type III | | |
|  | Wald Chi-Square | df | Sig. |
| (Intercept) | 55.899 | 1 | .000 |
| treatment_modalities2 | 34.301 | 1 | .000 |
| Dependent Variable: Outcome_recorded  Model: (Intercept), treatment_modalities2 | | | |

| **Parameter Estimates** | | | | | | | | | | |
| --- | --- | --- | --- | --- | --- | --- | --- | --- | --- | --- |
| Parameter | B | Std. Error | 95% Wald Confidence Interval | | Hypothesis Test | | | Exp(B) | 95% Wald Confidence Interval for Exp(B) | |
|  |  |  | Lower | Upper | Wald Chi-Square | df | Sig. |  | Lower | Upper |
| (Intercept) | -1.806 | .2639 | -2.323 | -1.288 | 46.816 | 1 | .000 | .164 | .098 | .276 |
| [treatment_modalities2=2] | 1.586 | .2708 | 1.055 | 2.117 | 34.301 | 1 | .000 | 4.885 | 2.873 | 8.306 |
| [treatment_modalities2=3] | 0^a^ | . | . | . | . | . | . | 1 | . | . |
| (Scale) | 1^b^ |  |  |  |  |  |  |  |  |  |
| Dependent Variable: Outcome_recorded  Model: (Intercept), treatment_modalities2 | | | | | | | | | | |
| a. Set to zero because this parameter is redundant. | | | | | | | | | | |
| b. Fixed at the displayed value. | | | | | | | | | | |

Marital_status_recorded2

| **Categorical Variable Information** | | | | |
| --- | --- | --- | --- | --- |
|  | | | N | Percent |
| Factor | Marital_status_recorded2 | Divorced | 17 | 12.2% |
|  |  | Married | 82 | 59.0% |
|  |  | Separated | 6 | 4.3% |
|  |  | Widowed | 14 | 10.1% |
|  |  | Single | 20 | 14.4% |
|  |  | Total | 139 | 100.0% |

| **Goodness of Fit^a^** | | | |
| --- | --- | --- | --- |
|  | Value | df | Value/df |
| Deviance | 92.789 | 134 | .692 |
| Scaled Deviance | 92.789 | 134 |  |
| Pearson Chi-Square | 74.000 | 134 | .552 |
| Scaled Pearson Chi-Square | 74.000 | 134 |  |
| Log Likelihood^b^ | -111.395 |  |  |
| Akaike's Information Criterion (AIC) | 232.789 |  |  |
| Finite Sample Corrected AIC (AICC) | 233.240 |  |  |
| Bayesian Information Criterion (BIC) | 247.462 |  |  |
| Consistent AIC (CAIC) | 252.462 |  |  |
| Dependent Variable: Outcome_recorded  Model: (Intercept), Marital_status_recorded2^a^ | | | |
| a. Information criteria are in smaller-is-better form. | | | |
| b. The full log likelihood function is displayed and used in computing information criteria. | | | |

| **Omnibus Test^a^** | | |
| --- | --- | --- |
| Likelihood Ratio Chi-Square | df | Sig. |
| 6.022 | 4 | .198 |
| Dependent Variable: Outcome_recorded  Model: (Intercept), Marital_status_recorded2^a^ | | |
| a. Compares the fitted model against the intercept-only model. | | |

| **Tests of Model Effects** | | | |
| --- | --- | --- | --- |
| Source | Type III | | |
|  | Wald Chi-Square | df | Sig. |
| (Intercept) | 40.858 | 1 | .000 |
| Marital_status_recorded2 | 7.350 | 4 | .119 |
| Dependent Variable: Outcome_recorded  Model: (Intercept), Marital_status_recorded2 | | | |

| **Parameter Estimates** | | | | | | | | | | |
| --- | --- | --- | --- | --- | --- | --- | --- | --- | --- | --- |
| Parameter | B | Std. Error | 95% Wald Confidence Interval | | Hypothesis Test | | | Exp(B) | 95% Wald Confidence Interval for Exp(B) | |
|  |  |  | Lower | Upper | Wald Chi-Square | df | Sig. |  | Lower | Upper |
| (Intercept) | -1.609 | .4472 | -2.486 | -.733 | 12.951 | 1 | .000 | .200 | .083 | .481 |
| [Marital_status_recorded2=1] | .568 | .5548 | -.519 | 1.655 | 1.048 | 1 | .306 | 1.765 | .595 | 5.235 |
| [Marital_status_recorded2=2] | 1.009 | .4583 | .111 | 1.908 | 4.851 | 1 | .028 | 2.744 | 1.118 | 6.737 |
| [Marital_status_recorded2=3] | 1.204 | .5323 | .161 | 2.247 | 5.116 | 1 | .024 | 3.333 | 1.174 | 9.462 |
| [Marital_status_recorded2=4] | .762 | .5434 | -.303 | 1.827 | 1.967 | 1 | .161 | 2.143 | .739 | 6.216 |
| [Marital_status_recorded2=5] | 0^a^ | . | . | . | . | . | . | 1 | . | . |
| (Scale) | 1^b^ |  |  |  |  |  |  |  |  |  |
| Dependent Variable: Outcome_recorded  Model: (Intercept), Marital_status_recorded2 | | | | | | | | | | |
| a. Set to zero because this parameter is redundant. | | | | | | | | | | |
| b. Fixed at the displayed value. | | | | | | | | | | |

CD4_Count (Binned)

| **Categorical Variable Information** | | | | |
| --- | --- | --- | --- | --- |
|  | | | N | Percent |
| Factor | CD4_Count (Binned) | < 200.0 | 95 | 68.3% |
|  |  | > = 200.0 | 44 | 31.7% |
|  |  | Total | 139 | 100.0% |

| **Goodness of Fit^a^** | | | |
| --- | --- | --- | --- |
|  | Value | df | Value/df |
| Deviance | 93.043 | 137 | .679 |
| Scaled Deviance | 93.043 | 137 |  |
| Pearson Chi-Square | 74.000 | 137 | .540 |
| Scaled Pearson Chi-Square | 74.000 | 137 |  |
| Log Likelihood^b^ | -111.521 |  |  |
| Akaike's Information Criterion (AIC) | 227.043 |  |  |
| Finite Sample Corrected AIC (AICC) | 227.131 |  |  |
| Bayesian Information Criterion (BIC) | 232.912 |  |  |
| Consistent AIC (CAIC) | 234.912 |  |  |
| Dependent Variable: Outcome_recorded  Model: (Intercept), CD4_Count (Binned)^a^ | | | |
| a. Information criteria are in smaller-is-better form. | | | |
| b. The full log likelihood function is displayed and used in computing information criteria. | | | |

| **Omnibus Test^a^** | | |
| --- | --- | --- |
| Likelihood Ratio Chi-Square | df | Sig. |
| 5.768 | 1 | .016 |
| Dependent Variable: Outcome_recorded  Model: (Intercept), CD4_Count (Binned)^a^ | | |
| a. Compares the fitted model against the intercept-only model. | | |

| **Tests of Model Effects** | | | |
| --- | --- | --- | --- |
| Source | Type III | | |
|  | Wald Chi-Square | df | Sig. |
| (Intercept) | 51.419 | 1 | .000 |
| CD4_Count (Binned) | 7.429 | 1 | .006 |
| Dependent Variable: Outcome_recorded  Model: (Intercept), CD4_Count (Binned) | | | |

| **Parameter Estimates** | | | | | | | | | | |
| --- | --- | --- | --- | --- | --- | --- | --- | --- | --- | --- |
| Parameter | B | Std. Error | 95% Wald Confidence Interval | | Hypothesis Test | | | Exp(B) | 95% Wald Confidence Interval for Exp(B) | |
|  |  |  | Lower | Upper | Wald Chi-Square | df | Sig. |  | Lower | Upper |
| (Intercept) | -1.299 | .2462 | -1.782 | -.817 | 27.854 | 1 | .000 | .273 | .168 | .442 |
| [CD4_Count (Binned)=1] | .716 | .2626 | .201 | 1.230 | 7.429 | 1 | .006 | 2.046 | 1.223 | 3.422 |
| [CD4_Count (Binned)=2] | 0^a^ | . | . | . | . | . | . | 1 | . | . |
| (Scale) | 1^b^ |  |  |  |  |  |  |  |  |  |
| Dependent Variable: Outcome_recorded  Model: (Intercept), CD4_Count (Binned) | | | | | | | | | | |
| a. Set to zero because this parameter is redundant. | | | | | | | | | | |
| b. Fixed at the displayed value. | | | | | | | | | | |

| **Categorical Variable Information** | | | | |
| --- | --- | --- | --- | --- |
|  | | | N | Percent |
| Factor | HIV_diagnosis_recorded | Known HIV patient | 101 | 72.7% |
|  |  | Newly diagnosed | 38 | 27.3% |
|  |  | Total | 139 | 100.0% |

| **Goodness of Fit^a^** | | | |
| --- | --- | --- | --- |
|  | Value | df | Value/df |
| Deviance | 98.563 | 137 | .719 |
| Scaled Deviance | 98.563 | 137 |  |
| Pearson Chi-Square | 74.000 | 137 | .540 |
| Scaled Pearson Chi-Square | 74.000 | 137 |  |
| Log Likelihood^b^ | -114.282 |  |  |
| Akaike's Information Criterion (AIC) | 232.563 |  |  |
| Finite Sample Corrected AIC (AICC) | 232.652 |  |  |
| Bayesian Information Criterion (BIC) | 238.432 |  |  |
| Consistent AIC (CAIC) | 240.432 |  |  |
| Dependent Variable: Outcome_recorded  Model: (Intercept), HIV_diagnosis_recorded^a^ | | | |
| a. Information criteria are in smaller-is-better form. | | | |
| b. The full log likelihood function is displayed and used in computing information criteria. | | | |

| **Omnibus Test^a^** | | |
| --- | --- | --- |
| Likelihood Ratio Chi-Square | df | Sig. |
| .248 | 1 | .619 |
| Dependent Variable: Outcome_recorded  Model: (Intercept), HIV_diagnosis_recorded^a^ | | |
| a. Compares the fitted model against the intercept-only model. | | |

| **Tests of Model Effects** | | | |
| --- | --- | --- | --- |
| Source | Type III | | |
|  | Wald Chi-Square | df | Sig. |
| (Intercept) | 54.029 | 1 | .000 |
| HIV_diagnosis_recorded | .430 | 1 | .512 |
| Dependent Variable: Outcome_recorded  Model: (Intercept), HIV_diagnosis_recorded | | | |

| **Parameter Estimates** | | | | | | | | | | |
| --- | --- | --- | --- | --- | --- | --- | --- | --- | --- | --- |
| Parameter | B | Std. Error | 95% Wald Confidence Interval | | Hypothesis Test | | | Exp(B) | 95% Wald Confidence Interval for Exp(B) | |
|  |  |  | Lower | Upper | Wald Chi-Square | df | Sig. |  | Lower | Upper |
| (Intercept) | -.865 | .1902 | -1.238 | -.492 | 20.678 | 1 | .000 | .421 | .290 | .611 |
| [HIV_diagnosis_recorded=1] | .142 | .2161 | -.282 | .565 | .430 | 1 | .512 | 1.152 | .754 | 1.760 |
| [HIV_diagnosis_recorded=2] | 0^a^ | . | . | . | . | . | . | 1 | . | . |
| (Scale) | 1^b^ |  |  |  |  |  |  |  |  |  |
| Dependent Variable: Outcome_recorded  Model: (Intercept), HIV_diagnosis_recorded | | | | | | | | | | |
| a. Set to zero because this parameter is redundant. | | | | | | | | | | |
| b. Fixed at the displayed value. | | | | | | | | | | |

WHO_clinical_stage_recorded

| **Categorical Variable Information** | | | | |
| --- | --- | --- | --- | --- |
|  | | | N | Percent |
| Factor | WHO_clinical_stage_recorded | Early stage (I & II) | 8 | 5.8% |
|  |  | Advanced stage (III & IV) | 131 | 94.2% |
|  |  | Total | 139 | 100.0% |

| **Continuous Variable Information** | | | | | | |
| --- | --- | --- | --- | --- | --- | --- |
|  | | N | Minimum | Maximum | Mean | Std. Deviation |
| Dependent Variable | Outcome_recorded | 139 | 0 | 1 | .47 | .501 |

| **Goodness of Fit^a^** | | | |
| --- | --- | --- | --- |
|  | Value | df | Value/df |
| Deviance | 98.402 | 137 | .718 |
| Scaled Deviance | 98.402 | 137 |  |
| Pearson Chi-Square | 74.000 | 137 | .540 |
| Scaled Pearson Chi-Square | 74.000 | 137 |  |
| Log Likelihood^b^ | -114.201 |  |  |
| Akaike's Information Criterion (AIC) | 232.402 |  |  |
| Finite Sample Corrected AIC (AICC) | 232.491 |  |  |
| Bayesian Information Criterion (BIC) | 238.271 |  |  |
| Consistent AIC (CAIC) | 240.271 |  |  |
| Dependent Variable: Outcome_recorded  Model: (Intercept), WHO_clinical_stage_recorded^a^ | | | |
| a. Information criteria are in smaller-is-better form. | | | |
| b. The full log likelihood function is displayed and used in computing information criteria. | | | |

| **Omnibus Test^a^** | | |
| --- | --- | --- |
| Likelihood Ratio Chi-Square | df | Sig. |
| .409 | 1 | .523 |
| Dependent Variable: Outcome_recorded  Model: (Intercept), WHO_clinical_stage_recorded^a^ | | |
| a. Compares the fitted model against the intercept-only model. | | |

| **Tests of Model Effects** | | | |
| --- | --- | --- | --- |
| Source | Type III | | |
|  | Wald Chi-Square | df | Sig. |
| (Intercept) | 18.619 | 1 | .000 |
| WHO_clinical_stage_recorded | 1.150 | 1 | .284 |
| Dependent Variable: Outcome_recorded  Model: (Intercept), WHO_clinical_stage_recorded | | | |

| **Parameter Estimates** | | | | | | | | | | |
| --- | --- | --- | --- | --- | --- | --- | --- | --- | --- | --- |
| Parameter | B | Std. Error | 95% Wald Confidence Interval | | Hypothesis Test | | | Exp(B) | 95% Wald Confidence Interval for Exp(B) | |
|  |  |  | Lower | Upper | Wald Chi-Square | df | Sig. |  | Lower | Upper |
| (Intercept) | -.781 | .0950 | -.967 | -.595 | 67.500 | 1 | .000 | .458 | .380 | .552 |
| [WHO_clinical_stage_recorded=1] | .311 | .2899 | -.257 | .879 | 1.150 | 1 | .284 | 1.365 | .773 | 2.409 |
| [WHO_clinical_stage_recorded=2] | 0^a^ | . | . | . | . | . | . | 1 | . | . |
| (Scale) | 1^b^ |  |  |  |  |  |  |  |  |  |
| Dependent Variable: Outcome_recorded  Model: (Intercept), WHO_clinical_stage_recorded | | | | | | | | | | |
| a. Set to zero because this parameter is redundant. | | | | | | | | | | |
| b. Fixed at the displayed value. | | | | | | | | | | |

ART_Defaulter_recorded

| **Categorical Variable Information** | | | | |
| --- | --- | --- | --- | --- |
|  | | | N | Percent |
| Factor | ART_Defaulter_recorded | Yes | 99 | 71.2% |
|  |  | No | 40 | 28.8% |
|  |  | Total | 139 | 100.0% |

| **Continuous Variable Information** | | | | | | |
| --- | --- | --- | --- | --- | --- | --- |
|  | | N | Minimum | Maximum | Mean | Std. Deviation |
| Dependent Variable | Outcome_recorded | 139 | 0 | 1 | .47 | .501 |

| **Goodness of Fit^a^** | | | |
| --- | --- | --- | --- |
|  | Value | df | Value/df |
| Deviance | 98.245 | 137 | .717 |
| Scaled Deviance | 98.245 | 137 |  |
| Pearson Chi-Square | 74.000 | 137 | .540 |
| Scaled Pearson Chi-Square | 74.000 | 137 |  |
| Log Likelihood^b^ | -114.122 |  |  |
| Akaike's Information Criterion (AIC) | 232.245 |  |  |
| Finite Sample Corrected AIC (AICC) | 232.333 |  |  |
| Bayesian Information Criterion (BIC) | 238.114 |  |  |
| Consistent AIC (CAIC) | 240.114 |  |  |
| Dependent Variable: Outcome_recorded  Model: (Intercept), ART_Defaulter_recorded^a^ | | | |
| a. Information criteria are in smaller-is-better form. | | | |
| b. The full log likelihood function is displayed and used in computing information criteria. | | | |

| **Omnibus Test^a^** | | |
| --- | --- | --- |
| Likelihood Ratio Chi-Square | df | Sig. |
| .567 | 1 | .452 |
| Dependent Variable: Outcome_recorded  Model: (Intercept), ART_Defaulter_recorded^a^ | | |
| a. Compares the fitted model against the intercept-only model. | | |

| **Tests of Model Effects** | | | |
| --- | --- | --- | --- |
| Source | Type III | | |
|  | Wald Chi-Square | df | Sig. |
| (Intercept) | 54.868 | 1 | .000 |
| ART_Defaulter_recorded | .949 | 1 | .330 |
| Dependent Variable: Outcome_recorded  Model: (Intercept), ART_Defaulter_recorded | | | |

| **Parameter Estimates** | | | | | | | | | | |
| --- | --- | --- | --- | --- | --- | --- | --- | --- | --- | --- |
| Parameter | B | Std. Error | 95% Wald Confidence Interval | | Hypothesis Test | | | Exp(B) | 95% Wald Confidence Interval for Exp(B) | |
|  |  |  | Lower | Upper | Wald Chi-Square | df | Sig. |  | Lower | Upper |
| (Intercept) | -.916 | .1936 | -1.296 | -.537 | 22.389 | 1 | .000 | .400 | .274 | .585 |
| [ART_Defaulter_recorded=2] | .213 | .2186 | -.216 | .642 | .949 | 1 | .330 | 1.237 | .806 | 1.899 |
| [ART_Defaulter_recorded=1] | 0^a^ | . | . | . | . | . | . | 1 | . | . |
| (Scale) | 1^b^ |  |  |  |  |  |  |  |  |  |
| Dependent Variable: Outcome_recorded  Model: (Intercept), ART_Defaulter_recorded | | | | | | | | | | |
| a. Set to zero because this parameter is redundant. | | | | | | | | | | |
| b. Fixed at the displayed value. | | | | | | | | | | |

**MULTIVARIABLE ANALYSIS**

| **Categorical Variable Information** | | | | |
| --- | --- | --- | --- | --- |
|  | | | N | Percent |
| Factor | Sex_recorded | Female | 81 | 58.3% |
|  |  | Male | 58 | 41.7% |
|  |  | Total | 139 | 100.0% |
|  | treatment_modalities2 | Amphotericin B based-Triple therapy | 66 | 47.5% |
|  |  | Fluconazole monotherapy | 73 | 52.5% |
|  |  | Total | 139 | 100.0% |
|  | Marital_status_recorded2 | Divorced | 17 | 12.2% |
|  |  | Married | 82 | 59.0% |
|  |  | Separated | 6 | 4.3% |
|  |  | Widowed | 14 | 10.1% |
|  |  | Single | 20 | 14.4% |
|  |  | Total | 139 | 100.0% |
|  | CD4_Count (Binned) | < 200.0 | 95 | 68.3% |
|  |  | > = 200.0 | 44 | 31.7% |
|  |  | Total | 139 | 100.0% |

| **Continuous Variable Information** | | | | | | |
| --- | --- | --- | --- | --- | --- | --- |
|  | | N | Minimum | Maximum | Mean | Std. Deviation |
| Dependent Variable | Outcome_recorded | 139 | 0 | 1 | .47 | .501 |

| **Goodness of Fit^a^** | | | |
| --- | --- | --- | --- |
|  | Value | df | Value/df |
| Deviance | 61.326 | 131 | .468 |
| Scaled Deviance | 61.326 | 131 |  |
| Pearson Chi-Square | 67.091 | 131 | .512 |
| Scaled Pearson Chi-Square | 67.091 | 131 |  |
| Log Likelihood^b^ | -95.663 |  |  |
| Akaike's Information Criterion (AIC) | 207.326 |  |  |
| Finite Sample Corrected AIC (AICC) | 208.434 |  |  |
| Bayesian Information Criterion (BIC) | 230.802 |  |  |
| Consistent AIC (CAIC) | 238.802 |  |  |
| Dependent Variable: Outcome_recorded  Model: (Intercept), Sex_recorded, treatment_modalities2, Marital_status_recorded2, CD4_Count (Binned)^a^ | | | |
| a. Information criteria are in smaller-is-better form. | | | |
| b. The full log likelihood function is displayed and used in computing information criteria. | | | |

| **Omnibus Test^a^** | | |
| --- | --- | --- |
| Likelihood Ratio Chi-Square | df | Sig. |
| 37.485 | 7 | .000 |
| Dependent Variable: Outcome_recorded  Model: (Intercept), Sex_recorded, treatment_modalities2, Marital_status_recorded2, CD4_Count (Binned)^a^ | | |
| a. Compares the fitted model against the intercept-only model. | | |

| **Tests of Model Effects** | | | |
| --- | --- | --- | --- |
| Source | Type III | | |
|  | Wald Chi-Square | df | Sig. |
| (Intercept) | 66.524 | 1 | .000 |
| Sex_recorded | 3.654 | 1 | .056 |
| treatment_modalities2 | 27.605 | 1 | .000 |
| Marital_status_recorded2 | 8.868 | 4 | .064 |
| CD4_Count (Binned) | 1.522 | 1 | .217 |
| Dependent Variable: Outcome_recorded  Model: (Intercept), Sex_recorded, treatment_modalities2, Marital_status_recorded2, CD4_Count (Binned) | | | |

| **Parameter Estimates** | | | | | | | | | | |
| --- | --- | --- | --- | --- | --- | --- | --- | --- | --- | --- |
| Parameter | B | Std. Error | 95% Wald Confidence Interval | | Hypothesis Test | | | Exp(B) | 95% Wald Confidence Interval for Exp(B) | |
|  |  |  | Lower | Upper | Wald Chi-Square | df | Sig. |  | Lower | Upper |
| (Intercept) | -2.609 | .4315 | -3.455 | -1.763 | 36.550 | 1 | .000 | .074 | .032 | .172 |
| [Sex_recorded=1] | .300 | .1567 | -.008 | .607 | 3.654 | 1 | .056 | 1.349 | .992 | 1.834 |
| [Sex_recorded=2] | 0^a^ | . | . | . | . | . | . | 1 | . | . |
| [treatment_modalities2=2] | 1.433 | .2728 | .899 | 1.968 | 27.605 | 1 | .000 | 4.192 | 2.456 | 7.155 |
| [treatment_modalities2=3] | 0^a^ | . | . | . | . | . | . | 1 | . | . |
| [Marital_status_recorded2=1] | .175 | .4256 | -.660 | 1.009 | .168 | 1 | .682 | 1.191 | .517 | 2.742 |
| [Marital_status_recorded2=2] | .656 | .3833 | -.095 | 1.408 | 2.932 | 1 | .087 | 1.928 | .909 | 4.086 |
| [Marital_status_recorded2=3] | .779 | .4316 | -.067 | 1.625 | 3.256 | 1 | .071 | 2.179 | .935 | 5.077 |
| [Marital_status_recorded2=4] | .321 | .4153 | -.492 | 1.135 | .599 | 1 | .439 | 1.379 | .611 | 3.112 |
| [Marital_status_recorded2=5] | 0^a^ | . | . | . | . | . | . | 1 | . | . |
| [CD4_Count (Binned)=1] | .261 | .2118 | -.154 | .676 | 1.522 | 1 | .217 | 1.299 | .857 | 1.967 |
| [CD4_Count (Binned)=2] | 0^a^ | . | . | . | . | . | . | 1 | . | . |
| (Scale) | 1^b^ |  |  |  |  |  |  |  |  |  |
| Dependent Variable: Outcome_recorded  Model: (Intercept), Sex_recorded, treatment_modalities2, Marital_status_recorded2, CD4_Count (Binned) | | | | | | | | | | |
| a. Set to zero because this parameter is redundant. | | | | | | | | | | |
| b. Fixed at the displayed value. | | | | | | | | | | |
