# Supplementary material for: In-hospital survival of adults with HIV-associated cryptococcal meningitis in Tanzania: A retrospective comparison of amphotericin B-based regimen and fluconazole monotherapy
Source: PLoS One. 2025 Dec 5;20(12):e0332786. doi: 10.1371/journal.pone.0332786 (PMC12680155; doi:10.1371/journal.pone.0332786)
Supplement: Attachment — Submitted filename: Response to Reviewers.docx [file pone.0332786.s003.docx]

**Response to reviewer comments – PLOS ONE**

**Study title:** In-Hospital Survival of Adults with HIV-Associated Cryptococcal Meningitis in Tanzania: A Retrospective Comparison of Amphotericin B-based Regimen and Fluconazole Monotherapy

**Reviewer 1**

**Comment 1:** In the background, the authors should give reasons as to why, despite the WHO Guidelines, approx half of the patients were receiving fluconazole mono therapy? This could be part of a selection bias.

**Response:** Thank you for the suggestions. We have revised the background and add the requested information to strengthen study rationale and to clear a doubt on selection bias

**Comment 2**: Equally explain bout the cost of care including the drugs and monitoring, are these provided for free by the government or are bought by the patients. This could equally affect the outcomes.

**Response:** Thank you for the suggestions. We have revised the background and add the requested information to strengthen study rationale and to help reader understand financing of the ART and opportunistic medications in low-middle income countries.
**Comment 3**: In the abstract, and manuscript, please remove the emphasis on "prevalence" as this is un-necessary, the title is clear on the objectives (In-hospital mortality and comparison of the treatment regimens).

**Response**: Well, noted. We have revised the whole manuscript and prevalence has been omitted accordingly throughout the manuscript.
**Comment 4**: In the methods and results, please restrict the study to the true study population, ie only those with a diagnosis of CCM.

**Response:** Thank you for the suggestion. We have revised study objective and re-analyse the data with the focus of only true study population i.e. People with HIV diagnosis admitted due to cryptococcal meningitis. Therefore, objective and results have been revised throughout the manuscript
